# Supplementary material for: ASIP, AHCY and ITCH Genes Are Associated with the Coat Color of Local Goats (Capra hircus) of Southwestern China
Source: Animals (Basel). 2025 Jun 23;15(13):1849. doi: 10.3390/ani15131849 (PMC12248594; doi:10.3390/ani15131849)
Supplement: Supplementary file 1 [file animals-15-01849-s001.zip › animals-3684878-supplementary.pdf]

animals MDPI

Table S1. List of Abbreviations.

Abbreviations	The full name of abbreviations
GWAS	Genome-wide association study
DZ	Dazu black goat
YD	Yudong black goat
BJ	Banjiao goat
HC	Hechuan white goat
CD	Chuandong white goat
YZ	Youzhou black goat
SNPs	Single nucleotide polymorphisms
MLM	Mixed linear model
PPI	Protein-protein interaction
GO	Gene ontology
CC	Cellular component
MF	Molecular function
BP	Biological process
KEGG	Kyoto encyclopedia of genes and genomes

Table S2. The genes associated with significant SNPs for black, white, and black-white coat colors in Chong-qing local goats.

**Chromosome**	**Position**	**Allele 1**	**Allele 2**	**P-value**	**Gene**	**Distance**
Chr1	21335233	C	T	1.55E-08	*SAMSN1*	53.14kb
Chr3	117692717	A	G	3.22E-08	*LOC108635666*	83.27kb
Chr3	37462925	A	C	3.27E-08	*KANK4*	130.47kb
Chr4	12607048	T	G	1.17E-10	*TPK1*	within
Chr4	12661864	T	G	4.06E-09	*TPK1*	within
Chr4	23786454	T	C	5.64E-09	*CHCHD3*	89.22kb
Chr4	12720187	C	A	1.23E-08	*TPK1*	within
Chr4	12644191	T	G	1.80E-08	*TPK1*	within
Chr4	12661035	T	C	2.03E-08	*TPK1*	within
Chr4	12656788	A	G	2.57E-08	*TPK1*	within
Chr4	12614701	T	A	3.29E-08	*TPK1*	within
Chr4	12599365	G	A	3.41E-08	*TPK1*	within
Chr4	12686567	A	T	4.18E-08	*TPK1*	within
Chr4	12662246	G	A	4.32E-08	*TPK1*	within
Chr4	12710068	G	A	4.35E-08	*TPK1*	within
Chr4	12735507	G	A	4.71E-08	*TPK1*	within
Chr4	12659781	A	G	5.20E-08	*TPK1*	within
Chr4	12590541	G	A	5.58E-08	*TPK1*	within
Chr5	31712612	C	T	2.32E-09	*ASB8*	within
Chr6	70585704	A	G	4.88E-11	*KIT*	125.52kb

Chr6	70585701	T	C	5.62E-11	*KIT*	125.53kb
Chr6	70584575	A	G	1.71E-09	*KIT*	126.66kb
Chr6	70583124	G	T	2.17E-09	*KIT*	128.11kb
Chr6	70585991	T	C	2.17E-09	*KIT*	125.24kb
Chr6	70590703	C	T	1.43E-08	*KIT*	120.53kb
Chr6	70582321	A	G	1.55E-08	*KIT*	128.91kb
Chr6	70584875	T	C	1.59E-08	*KIT*	126.36kb
Chr6	70586978	C	A	3.76E-08	*KIT*	124.25kb
Chr9	71271781	C	G	4.21E-08	*ADGB*	96.96kb
Chr9	71271674	A	C	5.55E-08	*ADGB*	96.85kb
Chr11	22236121	T	C	4.15E-09	*SLC8A1*	25.69kb
Chr11	22344118	A	G	3.56E-08	*SLC8A1*	within
Chr12	18575743	T	C	9.34E-09	*GPC5*	162.42kb
Chr12	54766891	T	C	9.70E-09	*FLT3*	30.16kb
Chr12	18565463	G	A	2.37E-08	*GPC5*	172.69kb
Chr12	54888680	A	C	3.12E-08	*PAN3*	within
Chr12	54813659	A	G	5.81E-08	*PAN3*	within
Chr12	54883890	T	C	5.92E-08	*PAN3*	within
Chr13	63248396	A	C	5.79E-28	*ASIP*	within
Chr13	63242361	T	C	3.72E-18	*ASIP*	within
Chr13	63248611	C	T	1.37E-17	*ASIP*	within
Chr13	63241024	C	T	7.53E-17	*ASIP*	within
Chr13	63242491	A	G	1.44E-16	*ASIP*	within
Chr13	63234020	C	T	6.36E-15	*ASIP*	within
Chr13	63238993	T	C	5.30E-14	*ASIP*	within
Chr13	63247390	T	C	5.85E-14	*ASIP*	within
Chr13	63249094	G	A	2.08E-11	*ASIP*	within
Chr13	63252814	A	G	4.65E-11	*ASIP*	3.27kb
Chr13	63232548	T	A	4.78E-11	*ASIP*	within
Chr13	63262679	G	T	1.81E-10	*AHCY*	1.38kb
Chr13	63235326	T	C	1.91E-10	*ASIP*	within
Chr13	63256334	A	G	1.91E-10	*ASIP*	6.79kb
Chr13	63232081	T	C	2.01E-10	*ASIP*	within
Chr13	63248986	A	G	2.41E-10	*ASIP*	within
Chr13	63257781	C	T	1.57E-09	*AHCY*	6.28kb
Chr13	63262957	G	C	3.45E-09	*AHCY*	1.10kb
Chr13	63254976	T	C	2.19E-08	*ASIP*	5.43kb
Chr14	73234424	C	T	2.46E-08	*PIGT*	within
Chr15	18123792	A	G	1.65E-08	*ABTB2*	within
Chr16	19158598	C	T	4.51E-10	*GPATCH2*	197.01kb
Chr16	62513233	G	A	5.94E-09	*NPL*	within
Chr19	60721191	T	C	2.63E-08	*LOC102178109*	within

Chr20	57583795	G	T	5.22E-09	*FBXL7*	40.01kb
Chr26	46431067	A	G	9.04E-10	*PCDH15*	within
Chr26	46432787	T	C	2.61E-08	*PCDH15*	within
Chr26	46432459	A	G	5.04E-08	*PCDH15*	within
Chr28	37008853	T	C	3.36E-08	*GNG4*	within
Chr29	35293239	C	T	5.53E-08	*TMEM45B*	within

Table S3. The genes associated with significant SNPs for black and white coat colors in Chongqing local goats.

Chromosome	Position	Allele 1	Allele 2	P-value	Gene	Distance
Chr2	122021266	C	T	1.18E-08	*PPP1R1C*	146.79kb
Chr2	122021266	C	T	1.18E-08	*PDE1A*	within
Chr10	74244578	C	T	5.07E-09	*RYR3*	within
Chr10	74244578	C	T	5.07E-09	*AVEN*	69.37kb
Chr10	74289507	T	C	5.60E-08	*RYR3*	within
Chr10	74289507	T	C	5.60E-08	*AVEN*	24.43kb
Chr10	74294113	T	C	4.27E-08	*RYR3*	within
Chr10	74294113	T	C	4.27E-08	*AVEN*	19.83kb
Chr10	74294864	G	C	2.02E-08	*RYR3*	within
Chr10	74294864	G	C	2.02E-08	*AVEN*	19.08kb
Chr10	74298281	C	T	5.60E-08	*RYR3*	within
Chr10	74298281	C	T	5.60E-08	*AVEN*	15.67kb
Chr10	74298282	T	C	5.60E-08	*RYR3*	within
Chr10	74298282	T	C	5.60E-08	*AVEN*	15.66kb
Chr13	63248396	C	A	1.02E-19	*EIF2S2*	165.96kb
Chr13	63248396	C	A	1.02E-19	*ASIP*	within
Chr13	63248396	C	A	1.02E-19	*AHCY*	15.66kb
Chr13	63248396	C	A	1.02E-19	*RALY*	194.59kb
Chr13	63248396	C	A	1.02E-19	*LOC102190531*	45.17kb
Chr13	63248396	C	A	1.02E-19	*ITCH*	114.71kb
Chr13	63243078	T	C	3.51E-19	*EIF2S2*	160.64kb
Chr13	63243078	T	C	3.51E-19	*ASIP*	within
Chr13	63243078	T	C	3.51E-19	*AHCY*	20.98kb
Chr13	63243078	T	C	3.51E-19	*RALY*	189.27kb
Chr13	63243078	T	C	3.51E-19	*LOC102190531*	39.86kb
Chr13	63243078	T	C	3.51E-19	*ITCH*	120.02kb
Chr13	63243202	A	T	4.56E-18	*EIF2S2*	160.07kb
Chr13	63243202	A	T	4.56E-18	*ASIP*	within
Chr13	63243202	A	T	4.56E-18	*AHCY*	20.86kb
Chr13	63243202	A	T	4.56E-18	*RALY*	189.40kb
Chr13	63243202	A	T	4.56E-18	*LOC102190531*	39.98kb
Chr13	63243202	A	T	4.56E-18	*ITCH*	119.90kb

Chr13	63242973	T	C	6.18E-17	*EIF2S2*	160.53kb
Chr13	63242973	T	C	6.18E-17	*ASIP*	6.57kb
Chr13	63242973	T	C	6.18E-17	*AHCY*	21.09kb
Chr13	63242973	T	C	6.18E-17	*RALY*	189.17kb
Chr13	63242973	T	C	6.18E-17	*LOC102190531*	39.75kb
Chr13	63242973	T	C	6.18E-17	*ITCH*	120.13kb
Chr13	63240981	G	A	5.79E-13	*EIF2S2*	158.54kb
Chr13	63240981	G	A	5.79E-13	*ASIP*	8.56kb
Chr13	63240981	G	A	5.79E-13	*AHCY*	23.08kb
Chr13	63240981	G	A	5.79E-13	*RALY*	187.18kb
Chr13	63240981	G	A	5.79E-13	*LOC102190531*	37.76kb
Chr13	63240981	G	A	5.79E-13	*ITCH*	122.12kb
Chr13	63242361	C	T	1.47E-12	*EIF2S2*	159.92kb
Chr13	63242361	C	T	1.47E-12	*ASIP*	7.18kb
Chr13	63242361	C	T	1.47E-12	*AHCY*	21.69kb
Chr13	63242361	C	T	1.47E-12	*RALY*	188.56kb
Chr13	63242361	C	T	1.47E-12	*LOC102190531*	39.14kb
Chr13	63242361	C	T	1.47E-12	*ITCH*	120.74kb
Chr13	63241845	T	C	1.76E-12	*EIF2S2*	159.40kb
Chr13	63241845	T	C	1.76E-12	*ASIP*	7.70kb
Chr13	63241845	T	C	1.76E-12	*AHCY*	22.21kb
Chr13	63241845	T	C	1.76E-12	*RALY*	188.04kb
Chr13	63241845	T	C	1.76E-12	*LOC102190531*	38.62kb
Chr13	63241845	T	C	1.76E-12	*ITCH*	121.26kb
Chr13	63238993	C	T	3.69E-12	*EIF2S2*	156.55kb
Chr13	63238993	C	T	3.69E-12	*ASIP*	within
Chr13	63238993	C	T	3.69E-12	*AHCY*	25.07kb
Chr13	63238993	C	T	3.69E-12	*RALY*	185.19kb
Chr13	63238993	C	T	3.69E-12	*LOC102190531*	35.77kb
Chr13	63238993	C	T	3.69E-12	*ITCH*	124.11kb
Chr13	63234020	T	C	1.61E-11	*EIF2S2*	151.58kb
Chr13	63234020	T	C	1.61E-11	*ASIP*	5.31kb
Chr13	63234020	T	C	1.61E-11	*AHCY*	45.80kb
Chr13	63234020	T	C	1.61E-11	*RALY*	180.21kb
Chr13	63234020	T	C	1.61E-11	*LOC102190531*	30.79kb
Chr13	63234020	T	C	1.61E-11	*ITCH*	129.08kb
Chr13	63235326	C	T	4.50E-11	*EIF2S2*	152.88kb
Chr13	63235326	C	T	4.50E-11	*ASIP*	6.62kb
Chr13	63235326	C	T	4.50E-11	*AHCY*	28.73kb
Chr13	63235326	C	T	4.50E-11	*RALY*	181.52kb
Chr13	63235326	C	T	4.50E-11	*LOC102190531*	32.10kb
Chr13	63235326	C	T	4.50E-11	*ITCH*	127.78kb

Chr13	63241024	T	C	1.06E-10	*EIF2S2*	158.58kb
Chr13	63241024	T	C	1.06E-10	*ASIP*	8.52kb
Chr13	63241024	T	C	1.06E-10	*AHCY*	23.04kb
Chr13	63241024	T	C	1.06E-10	*RALY*	187.22kb
Chr13	63241024	T	C	1.06E-10	*LOC102190531*	37.80kb
Chr13	63241024	T	C	1.06E-10	*ITCH*	122.08kb
Chr13	63248986	G	A	1.26E-10	*EIF2S2*	166.54kb
Chr13	63248986	G	A	1.26E-10	*ASIP*	0.56kb
Chr13	63248986	G	A	1.26E-10	*AHCY*	15.07kb
Chr13	63248986	G	A	1.26E-10	*RALY*	195.18kb
Chr13	63248986	G	A	1.26E-10	*LOC102190531*	45.76kb
Chr13	63248986	G	A	1.26E-10	*ITCH*	114.12kb
Chr13	63242472	G	T	4.82E-10	*EIF2S2*	160.03kb
Chr13	63242472	G	T	4.82E-10	*ASIP*	7.07kb
Chr13	63242472	G	T	4.82E-10	*AHCY*	21.59kb
Chr13	63242472	G	T	4.82E-10	*RALY*	188.67kb
Chr13	63242472	G	T	4.82E-10	*LOC102190531*	39.25kb
Chr13	63242472	G	T	4.82E-10	*ITCH*	120.63kb
Chr13	63247390	C	T	1.17E-09	*EIF2S2*	164.95kb
Chr13	63247390	C	T	1.17E-09	*ASIP*	2.15kb
Chr13	63247390	C	T	1.17E-09	*AHCY*	16.67kb
Chr13	63247390	C	T	1.17E-09	*RALY*	193.58kb
Chr13	63247390	C	T	1.17E-09	*LOC102190531*	44.17kb
Chr13	63247390	C	T	1.17E-09	*ITCH*	115.71kb
Chr13	63242491	G	A	1.36E-09	*EIF2S2*	160.05kb
Chr13	63242491	G	A	1.36E-09	*ASIP*	7.05kb
Chr13	63242491	G	A	1.36E-09	*AHCY*	21.57kb
Chr13	63242491	G	A	1.36E-09	*RALY*	188.69kb
Chr13	63242491	G	A	1.36E-09	*LOC102190531*	39.27kb
Chr13	63242491	G	A	1.36E-09	*ITCH*	120.06kb
Chr13	63252814	G	A	2.77E-09	*EIF2S2*	170.37kb
Chr13	63252814	G	A	2.77E-09	*ASIP*	3.27kb
Chr13	63252814	G	A	2.77E-09	*AHCY*	11.25kb
Chr13	63252814	G	A	2.77E-09	*RALY*	199.01kb
Chr13	63252814	G	A	2.77E-09	*LOC102190531*	49.59kb
Chr13	63252814	G	A	2.77E-09	*ITCH*	110.29kb
Chr13	63235341	G	A	8.42E-09	*EIF2S2*	152.89kb
Chr13	63235341	G	A	8.42E-09	*ASIP*	6.63kb
Chr13	63235341	G	A	8.42E-09	*AHCY*	28.72kb
Chr13	63235341	G	A	8.42E-09	*RALY*	181.54kb
Chr13	63235341	G	A	8.42E-09	*LOC102190531*	32.12kb
Chr13	63235341	G	A	8.42E-09	*ITCH*	127.76kb

Chr13	63271556	G	A	1.83E-08	*EIF2S2*	189.11kb
Chr13	63271556	G	A	1.83E-08	*ASIP*	22.01kb
Chr13	63271556	G	A	1.83E-08	*AHCY*	within
Chr13	63271556	G	A	1.83E-08	*DYNLRB1*	193.99kb
Chr13	63271556	G	A	1.83E-08	*LOC102190531*	68.33kb
Chr13	63271556	G	A	1.83E-08	*ITCH*	91.55kb
Chr13	63262679	G	T	4.71E-08	*EIF2S2*	180.24kb
Chr13	63262679	G	T	4.71E-08	*ASIP*	13.14kb
Chr13	63262679	G	T	4.71E-08	*AHCY*	1.38kb
Chr13	63262679	G	T	4.71E-08	*LOC102190531*	89.71kb
Chr13	63262679	G	T	4.71E-08	*ITCH*	100.43kb
Chr13	63248611	T	C	5.65E-08	*EIF2S2*	166.17kb
Chr13	63248611	T	C	5.65E-08	*ASIP*	0.93kb
Chr13	63248611	T	C	5.65E-08	*AHCY*	15.45kb
Chr13	63248611	T	C	5.65E-08	*RALY*	194.81kb
Chr13	63248611	T	C	5.65E-08	*LOC102190531*	45.39kb
Chr13	63248611	T	C	5.65E-08	*ITCH*	114.49kb
Chr15	56626730	A	G	2.32E-08	*LOC108637682*	7.60kb
Chr15	56626730	A	G	2.32E-08	*LOC102182151*	76.60kb
Chr15	56626730	A	G	2.32E-08	*CADM1*	within
Chr18	9469303	G	A	3.01E-09	*LOC102181146*	86.51kb
Chr18	9469303	G	A	3.01E-09	*GAN*	138.86kb
Chr18	9469303	G	A	3.01E-09	*PLCG2*	172.37kb
Chr18	9469303	G	A	3.01E-09	*CMIP*	within

Table S4. The genes associated with significant SNPs for black and black-white coat color in Chongqing local goats.

Chromosome	Position	Allele 1	Allele 2	P-value	Gene	Distance
Chr2	20786644	T	C	1.02E-09	*IRS1*	within
Chr2	20786644	T	C	1.02E-09	*RHBDD1*	86.65kb
Chr5	61149040	A	G	7.20E-10	*LOC108636107*	3.66kb
Chr5	66270314	G	T	3.05E-10	*TRNAW-CCA-25*	49.73kb
Chr5	66270314	G	T	3.05E-10	*LOC102169908*	167.72kb
Chr5	66270314	G	T	3.05E-10	*LOC102179170*	within
Chr5	66270314	G	T	3.05E-10	*HSP90B1*	17.71kb
Chr5	66270314	G	T	3.05E-10	*TDG*	53.60kb
Chr5	66270314	G	T	3.05E-10	*GLT8D2*	75.10kb
Chr5	66270314	G	T	3.05E-10	*HCFC2*	127.09kb
Chr5	66270314	G	T	3.05E-10	*NFYB*	183.90kb
Chr5	66270314	G	T	3.05E-10	*NT5DC3*	66.32kb
Chr5	66270314	G	T	3.05E-10	*C5H12orf73*	40.38kb

Chr5	66270314	G	T	3.05E-10	*STAB2*	135.63kb
Chr7	95092759	G	A	1.94E-10	*S1PR5*	184.07kb
Chr7	95092759	G	A	1.94E-10	*CDKN2D*	139.40kb
Chr7	95092759	G	A	1.94E-10	*TMED1*	69.80kb
Chr7	95092759	G	A	1.94E-10	*YIPF2*	133.65kb
Chr7	95092759	G	A	1.94E-10	*C7H19orf52*	139.35kb
Chr7	95092759	G	A	1.94E-10	*LOC102186002*	151.45kb
Chr7	95092759	G	A	1.94E-10	*LOC106502300*	156.50kb
Chr7	95092759	G	A	1.94E-10	*ATG4D*	152.17kb
Chr7	95092759	G	A	1.94E-10	*KRI1*	143.04kb
Chr7	95092759	G	A	1.94E-10	*AP1M2*	127.85kb
Chr7	95092759	G	A	1.94E-10	*SLC44A2*	84.44kb
Chr7	95092759	G	A	1.94E-10	*QTRT1*	30.67kb
Chr7	95092759	G	A	1.94E-10	*C7H19orf38*	80.45kb
Chr7	95092759	G	A	1.94E-10	*CARM1*	92.29kb
Chr7	95092759	G	A	1.94E-10	*KEAP1*	194.11kb
Chr7	95092759	G	A	1.94E-10	*ILF3*	48.93kb
Chr7	95092759	G	A	1.94E-10	*DNM2*	within
Chr7	95096084	T	G	1.91E-09	*DNM2*	within
Chr7	96661838	A	G	1.02E-09	*NDUFB7*	35.96kb
Chr7	96661838	A	G	1.02E-09	*TRNAC-GCA-122*	8.61kb
Chr7	96661838	A	G	1.02E-09	*PTGER1*	31.47kb
Chr7	96661838	A	G	1.02E-09	*DDX39A*	76.14kb
Chr7	96661838	A	G	1.02E-09	*ADGRE3*	88.91kb
Chr7	96661838	A	G	1.02E-09	*TECR*	7.69kb
Chr7	96661838	A	G	1.02E-09	*LOC102172137*	4.49kb
Chr7	96661838	A	G	1.02E-09	*DNAJB1*	1.26kb
Chr7	96661838	A	G	1.02E-09	*GIPC1*	16.54kb
Chr7	96661838	A	G	1.02E-09	*PKN1*	35.36kb
Chr7	96661838	A	G	1.02E-09	*ADGRE5*	85.04kb
Chr7	96661838	A	G	1.02E-09	*CLEC17A*	52.79kb
Chr8	102968848	G	A	1.19E-12	*ZNF618*	within
Chr8	102974356	T	C	1.04E-09	*ZNF618*	within
Chr8	102980454	T	C	1.19E-12	*KIF12*	58.03kb
Chr8	102980454	T	C	1.19E-12	*ZNF618*	within
Chr8	103037495	A	G	1.72E-10	*KIF12*	0.99kb
Chr8	103037495	A	G	1.72E-10	*ZNF618*	32.29kb
Chr8	103037495	A	G	1.72E-10	*AMBP*	13.15kb
Chr8	103037495	A	G	1.72E-10	*COL27A1*	59.22kb
Chr9	73390777	T	C	9.47E-11	*TRNAC-GCA-139*	156.62kb
Chr9	73390777	T	C	9.47E-11	*UST*	97.33kb
Chr9	73390777	T	C	9.47E-11	*TAB2*	136.63kb

Chr9	76069485	A	T	3.58E-10	*CCDC170*	155.19kb
Chr9	76069485	A	T	3.58E-10	*ESR1*	within
Chr10	47087471	C	T	3.01E-11	*UNC13C*	within
Chr10	47347022	A	G	4.58E-10	*UNC13C*	within
Chr12	83631345	A	G	1.40E-12	*DIAPH3*	167.31kb
Chr13	63243202	A	T	8.77E-71	*ASIP*	within
Chr13	63242973	T	C	5.25E-46	*ASIP*	within
Chr13	63243078	T	C	1.38E-30	*ASIP*	within
Chr13	63243078	T	C	1.38E-30	*AHCY*	20.98kb
Chr13	63232101	C	T	1.23E-23	*EIF2S2*	149.66kb
Chr13	63232101	C	T	1.23E-23	*ASIP*	within
Chr13	63232101	C	T	1.23E-23	*RALY*	178.30kb
Chr13	63232101	C	T	1.23E-23	*LOC102190531*	28.88kb
Chr13	63242472	G	T	1.11E-22	*ASIP*	within
Chr13	63241845	T	C	5.02E-22	*ASIP*	within
Chr13	63240981	G	A	3.26E-15	*EIF2S2*	158.54kb
Chr13	63240981	G	A	3.26E-15	*ASIP*	within
Chr13	63248396	C	A	2.12E-14	*ASIP*	within
Chr13	63248396	C	A	2.12E-14	*AHCY*	15.66kb
Chr13	63248396	C	A	2.12E-14	*ITCH*	114.71kb
Chr13	63243753	T	A	6.97E-12	*ASIP*	within
Chr13	61725216	T	C	1.37E-11	*BPIFB4*	within
Chr13	63242361	C	T	4.74E-11	*ASIP*	within
Chr13	67454791	A	G	3.50E-10	*FAM83D*	125.68kb
Chr13	67454791	A	G	3.50E-10	*PPP1R16B*	151.62kb
Chr13	67454791	A	G	3.50E-10	*DHX35*	49.14kb
Chr13	61713079	G	A	6.44E-10	*BPIFB2*	62.92kb
Chr13	61713079	G	A	6.44E-10	*BPIFB6*	40.50kb
Chr13	61713079	G	A	6.44E-10	*BPIFB3*	16.59kb
Chr13	61713079	G	A	6.44E-10	*BPIFB4*	within
Chr13	61713079	G	A	6.44E-10	*SUN5*	86.12kb
Chr13	61469024	G	T	9.11E-10	*LOC102184536*	77.44kb
Chr13	61469024	G	T	9.11E-10	*TRNAR-CCU-14*	41.63kb
Chr13	61469024	G	T	9.11E-10	*COMMD7*	13.55kb
Chr13	61469024	G	T	9.11E-10	*NOL4L*	133.98kb
Chr13	61469024	G	T	9.11E-10	*DNMT3B*	within
Chr13	61736057	A	G	9.98E-10	*LOC106502777*	18.23kb
Chr13	67500652	A	G	1.23E-09	*TRNAE-UUC-58*	50.34kb
Chr13	67500652	A	G	1.23E-09	*FAM83D*	171.54kb
Chr13	67500652	A	G	1.23E-09	*DHX35*	95.00kb
Chr13	61482022	A	G	1.40E-09	*LOC102184536*	90.44kb
Chr13	61482022	A	G	1.40E-09	*MAPRE1*	26.44kb

Chr13	61482022	A	G	1.40E-09	*DNMT3B*	within
Chr13	61718283	C	A	1.84E-09	*BPIFB2*	68.13kb
Chr13	61718283	C	A	1.84E-09	*BPIFB6*	45.70kb
Chr13	61718283	C	A	1.84E-09	*BPIFB3*	21.79kb
Chr13	61718283	C	A	1.84E-09	*BPIFB4*	within
Chr14	93093692	A	T	8.03E-10	*RIMS1*	within
Chr15	21950206	T	C	1.82E-09	*ARL14EP*	100.92kb
Chr15	21951120	C	T	3.96E-11	*FSHB*	29.18kb
Chr17	61098631	C	T	6.70E-12	*NR3C2*	within
Chr17	64963348	G	A	2.69E-11	*GATB*	105.35kb
Chr17	64968290	A	G	2.70E-11	*GATB*	110.29kb
Chr17	64982947	G	C	2.80E-11	*GATB*	124.95kb
Chr17	64952791	C	A	2.01E-10	*GATB*	94.79kb
Chr17	64952791	C	A	2.01E-10	*FAM160A1*	196.83kb
Chr17	64968538	A	G	3.41E-10	*GATB*	110.54kb
Chr17	764512	G	A	6.15E-10	*LOC108637841*	185.97kb
Chr17	764512	G	A	6.15E-10	*LOC102172893*	147.84kb
Chr17	764512	G	A	6.15E-10	*LOC102175702*	119.25kb
Chr17	764512	G	A	6.15E-10	*YWHAH*	58.96kb
Chr17	764512	G	A	6.15E-10	*LOC102175148*	67.56kb
Chr17	764512	G	A	6.15E-10	*SLC5A1*	6.42kb
Chr17	764512	G	A	6.15E-10	*PRR14L*	169.39kb
Chr17	764512	G	A	6.15E-10	*LOC102176353*	116.48kb
Chr17	764512	G	A	6.15E-10	*DEPDC5*	94.01kb
Chr18	5694474	C	G	5.10E-10	*SYCE1L*	43.63kb
Chr18	5694474	C	G	5.10E-10	*LOC108637953*	36.99kb
Chr18	5694474	C	G	5.10E-10	*MON1B*	54.80kb
Chr18	5694474	C	G	5.10E-10	*ADAMTS18*	44.92kb
Chr20	64134555	A	G	6.59E-10	*SEMA5A*	within
Chr20	64157934	T	C	6.59E-10	*SEMA5A*	within
Chr23	7825060	G	C	7.35E-11	*CD83*	137.14kb
Chr26	11989116	C	G	1.00E-09	*SEC23IP*	0.26kb
Chr26	11989116	C	G	1.00E-09	*MCMBP*	14.76kb
Chr26	11989116	C	G	1.00E-09	*INPP5F*	53.79kb
Chr27	3099646	T	C	3.93E-10	*LOC102174361*	within
Chr27	3105109	A	G	3.93E-10	*LOC102174361*	within
Chr27	3397311	T	C	9.37E-10	*NKIRAS1*	187.88kb
Chr27	3397311	T	C	9.37E-10	*LOC102173916*	115.07kb
Chr27	3397311	T	C	9.37E-10	*LOC102174361*	64.70kb

Table S5. The genes associated with significant SNPs for white and black-white coat colors in Chongqing local goats.

Chromosome	Position	Allele 1	Allele 2	P-value	Gene	Distance
Chr1	51340319	C	G	3.76E-08	*LOC108636831*	within
Chr1	51340319	C	G	3.76E-08	*KCNJ6*	16.24kb
Chr4	88122021	T	A	1.54E-08	*LOC108635917*	92.85kb
Chr4	88122021	T	A	1.54E-08	*LOC108635918*	37.15kb
Chr4	88122021	T	A	1.54E-08	*DBF4*	149.48kb
Chr4	88122021	T	A	1.54E-08	*LOC102171502*	134.61kb
Chr4	88122021	T	A	1.54E-08	*LOC102176015*	67.89kb
Chr4	88122021	T	A	1.54E-08	*LOC102174202*	within
Chr11	54447234	C	T	4.62E-08	*CTNNA2*	within
Chr13	63216991	T	C	8.19E-21	*EIF2S2*	134.55kb
Chr13	63216991	T	C	8.19E-21	*ASIP*	11.72kb
Chr13	63216991	T	C	8.19E-21	*AHCY*	47.07kb
Chr13	63216991	T	C	8.19E-21	*RALY*	163.19kb
Chr13	63216991	T	C	8.19E-21	*LOC102190531*	13.77kb
Chr13	63216991	T	C	8.19E-21	*ITCH*	146.11kb
Chr13	63227787	C	T	1.94E-18	*EIF2S2*	145.35kb
Chr13	63227787	C	T	1.94E-18	*ASIP*	0.92kb
Chr13	63227787	C	T	1.94E-18	*AHCY*	36.27kb
Chr13	63227787	C	T	1.94E-18	*RALY*	173.98kb
Chr13	63227787	C	T	1.94E-18	*LOC102190531*	24.57kb
Chr13	63227787	C	T	1.94E-18	*ITCH*	136.32kb
Chr13	63229219	G	C	2.78E-18	*EIF2S2*	146.78kb
Chr13	63229219	G	C	2.78E-18	*ASIP*	within
Chr13	63229219	G	C	2.78E-18	*AHCY*	34.84kb
Chr13	63229219	G	C	2.78E-18	*RALY*	175.21kb
Chr13	63229219	G	C	2.78E-18	*LOC102190531*	25.99kb
Chr13	63229219	G	C	2.78E-18	*ITCH*	133.89kb
Chr13	63228709	A	G	9.34E-17	*EIF2S2*	146.27kb
Chr13	63228709	A	G	9.34E-17	*ASIP*	within
Chr13	63228709	A	G	9.34E-17	*AHCY*	35.35kb
Chr13	63228709	A	G	9.34E-17	*RALY*	174.90kb
Chr13	63228709	A	G	9.34E-17	*LOC102190531*	25.49kb
Chr13	63228709	A	G	9.34E-17	*ITCH*	134.39kb
Chr13	63225873	A	T	1.69E-16	*EIF2S2*	143.43kb
Chr13	63225873	A	T	1.69E-16	*ASIP*	2.84kb
Chr13	63225873	A	T	1.69E-16	*AHCY*	38.17kb
Chr13	63225873	A	T	1.69E-16	*RALY*	172.01kb
Chr13	63225873	A	T	1.69E-16	*LOC102190531*	22.65kb
Chr13	63225873	A	T	1.69E-16	*ITCH*	137.23kb

Chr13	63224594	C	T	2.25E-16	*EIF2S2*	142.15kb
Chr13	63224594	C	T	2.25E-16	*ASIP*	4.12kb
Chr13	63224594	C	T	2.25E-16	*AHCY*	39.47kb
Chr13	63224594	C	T	2.25E-16	*RALY*	170.79kb
Chr13	63224594	C	T	2.25E-16	*LOC102190531*	21.37kb
Chr13	63224594	C	T	2.25E-16	*ITCH*	138.51kb
Chr13	63219374	C	T	3.78E-16	*EIF2S2*	136.93kb
Chr13	63219374	C	T	3.78E-16	*ASIP*	9.34kb
Chr13	63219374	C	T	3.78E-16	*AHCY*	44.69kb
Chr13	63219374	C	T	3.78E-16	*RALY*	165.57kb
Chr13	63219374	C	T	3.78E-16	*LOC102190531*	16.15kb
Chr13	63219374	C	T	3.78E-16	*ITCH*	143.73kb
Chr13	63230527	T	C	1.41E-14	*EIF2S2*	148.09kb
Chr13	63230527	T	C	1.41E-14	*ASIP*	within
Chr13	63230527	T	C	1.41E-14	*AHCY*	33.53kb
Chr13	63230527	T	C	1.41E-14	*RALY*	176.72kb
Chr13	63230527	T	C	1.41E-14	*LOC102190531*	27.31kb
Chr13	63230527	T	C	1.41E-14	*ITCH*	132.58kb
Chr13	63223175	T	A	3.49E-14	*EIF2S2*	140.73kb
Chr13	63223175	T	A	3.49E-14	*ASIP*	5.53kb
Chr13	63223175	T	A	3.49E-14	*AHCY*	40.88kb
Chr13	63223175	T	A	3.49E-14	*RALY*	169.37kb
Chr13	63223175	T	A	3.49E-14	*LOC102190531*	19.95kb
Chr13	63223175	T	A	3.49E-14	*ITCH*	139.93kb
Chr13	63224883	T	A	5.57E-14	*EIF2S2*	142.44kb
Chr13	63224883	T	A	5.57E-14	*ASIP*	3.83kb
Chr13	63224883	T	A	5.57E-14	*AHCY*	39.18kb
Chr13	63224883	T	A	5.57E-14	*RALY*	171.08kb
Chr13	63224883	T	A	5.57E-14	*LOC102190531*	21.67kb
Chr13	63224883	T	A	5.57E-14	*ITCH*	138.22kb
Chr13	63227239	G	C	1.85E-13	*EIF2S2*	144.79kb
Chr13	63227239	G	C	1.85E-13	*ASIP*	1.47kb
Chr13	63227239	G	C	1.85E-13	*AHCY*	36.82kb
Chr13	63227239	G	C	1.85E-13	*RALY*	173.43kb
Chr13	63227239	G	C	1.85E-13	*LOC102190531*	24.02kb
Chr13	63227239	G	C	1.85E-13	*ITCH*	135.87kb
Chr13	63380810	G	C	3.70E-13	*MAP1LC3A*	118.13kb
Chr13	63380810	G	C	3.70E-13	*ASIP*	131.27kb
Chr13	63380810	G	C	3.70E-13	*AHCY*	100.99kb
Chr13	63380810	G	C	3.70E-13	*DYNLRB1*	84.74kb
Chr13	63380810	G	C	3.70E-13	*LOC102190531*	177.59kb
Chr13	63380810	G	C	3.70E-13	*ITCH*	within

Chr13	63380810	G	C	3.70E-13	*PIGU*	120.11kb
Chr13	63220215	A	T	5.63E-11	*EIF2S2*	137.77kb
Chr13	63220215	A	T	5.63E-11	*ASIP*	8.49kb
Chr13	63220215	A	T	5.63E-11	*AHCY*	43.84kb
Chr13	63220215	A	T	5.63E-11	*RALY*	166.41kb
Chr13	63220215	A	T	5.63E-11	*LOC102190531*	16.99kb
Chr13	63220215	A	T	5.63E-11	*ITCH*	143.89kb
Chr13	63370756	G	A	4.77E-10	*MAP1LC3A*	128.18kb
Chr13	63370756	G	A	4.77E-10	*ASIP*	121.21kb
Chr13	63370756	G	A	4.77E-10	*AHCY*	90.94kb
Chr13	63370756	G	A	4.77E-10	*DYNLRB1*	94.79kb
Chr13	63370756	G	A	4.77E-10	*LOC102190531*	167.53kb
Chr13	63370756	G	A	4.77E-10	*ITCH*	within
Chr13	63370756	G	A	4.77E-10	*PIGU*	130.16kb
Chr13	63379243	C	T	1.11E-08	*MAP1LC3A*	119.69kb
Chr13	63379243	C	T	1.11E-08	*ASIP*	129.70kb
Chr13	63379243	C	T	1.11E-08	*AHCY*	99.42kb
Chr13	63379243	C	T	1.11E-08	*DYNLRB1*	86.31kb
Chr13	63379243	C	T	1.11E-08	*LOC102190531*	176.02kb
Chr13	63379243	C	T	1.11E-08	*ITCH*	within
Chr13	63379243	C	T	1.11E-08	*PIGU*	121.68kb
Chr13	63233047	G	T	1.18E-08	*EIF2S2*	150.61kb
Chr13	63233047	G	T	1.18E-08	*ASIP*	within
Chr13	63233047	G	T	1.18E-08	*AHCY*	31.01kb
Chr13	63233047	G	T	1.18E-08	*RALY*	179.24kb
Chr13	63233047	G	T	1.18E-08	*LOC102190531*	29.83kb
Chr13	63233047	G	T	1.18E-08	*ITCH*	130.06kb
Chr13	63366961	G	A	1.25E-08	*MAP1LC3A*	131.98kb
Chr13	63366961	G	A	1.25E-08	*ASIP*	117.42kb
Chr13	63366961	G	A	1.25E-08	*AHCY*	87.14kb
Chr13	63366961	G	A	1.25E-08	*DYNLRB1*	98.58kb
Chr13	63366961	G	A	1.25E-08	*LOC102190531*	163.74kb
Chr13	63366961	G	A	1.25E-08	*ITCH*	within
Chr13	63366961	G	A	1.25E-08	*PIGU*	133.96kb
Chr13	63374419	C	T	5.52E-08	*MAP1LC3A*	124.52kb
Chr13	63374419	C	T	5.52E-08	*ASIP*	124.88kb
Chr13	63374419	C	T	5.52E-08	*AHCY*	94.59kb
Chr13	63374419	C	T	5.52E-08	*DYNLRB1*	91.13kb
Chr13	63374419	C	T	5.52E-08	*LOC102190531*	171.19kb
Chr13	63374419	C	T	5.52E-08	*ITCH*	within
Chr13	63374419	C	T	5.52E-08	*PIGU*	126.49kb
Chr16	60333364	C	A	1.84E-08	*ACBD6*	68.58kb

Chr16	60333364	C	A	1.84E-08	*XPR1*	65.99kb
Chr25	39828132	G	A	2.59E-12	*ACTB*	166.06kb
Chr25	39828132	G	A	2.59E-12	*LOC102180279*	22.82kb
Chr25	39828132	G	A	2.59E-12	*PAPOLB*	170.06kb
Chr25	39828132	G	A	2.59E-12	*FBXL18*	132.33kb
Chr25	39828132	G	A	2.59E-12	*TNRC18*	4.54kb
Chr25	39828132	G	A	2.59E-12	*WIPI2*	41.88kb
Chr25	39828132	G	A	2.59E-12	*LOC106503599*	89.38kb
Chr25	39828132	G	A	2.59E-12	*SLC29A4*	within
Chr26	35505423	G	T	1.23E-08	*LOC108634008*	104.32kb
Chr26	35505423	G	T	1.23E-08	*LOC102185056*	123.82kb
Chr26	35505423	G	T	1.23E-08	*LOC102185708*	70.77kb
Chr26	35505423	G	T	1.23E-08	*HELLS*	52.39kb
Chr26	35505423	G	T	1.23E-08	*LOC102186740*	107.33kb
Chr26	35505423	G	T	1.23E-08	*LOC102185739*	within
Chr26	35505423	G	T	1.23E-08	*TBC1D12*	84.97kb

Table S6. Functional annotation of significant SNPs within the ASIP gene region associated with coat color in Chongqing goats.

Chromosome	Position	REF	ALT	Annotation	Annotation_Impact
Chr13	63228709	G	A	5_prime_UTR_variant	non_coding_transcript_variant
Chr13	63230527	T	C	intron_variant	non_coding_transcript_variant
Chr13	63232081	C	T	intron_variant	non_coding_transcript_variant
Chr13	63232101	T	C	intron_variant	non_coding_transcript_variant
Chr13	63232548	A	T	intron_variant	non_coding_transcript_variant
Chr13	63233047	T	G	intron_variant	non_coding_transcript_variant
Chr13	63234020	T	C	intron_variant	non_coding_transcript_variant
Chr13	63235326	C	T	intron_variant	non_coding_transcript_variant
Chr13	63238993	C	T	intron_variant	non_coding_transcript_variant
Chr13	63240981	A	G	intron_variant	non_coding_transcript_variant
Chr13	63241024	T	C	intron_variant	non_coding_transcript_variant
Chr13	63241845	C	T	intron_variant	non_coding_transcript_variant
Chr13	63242361	C	T	intron_variant	non_coding_transcript_variant
Chr13	63242472	T	G	intron_variant	non_coding_transcript_variant
Chr13	63242491	G	A	intron_variant	non_coding_transcript_variant
Chr13	63242973	C	T	intron_variant	non_coding_transcript_variant
Chr13	63243078	C	T	intron_variant	non_coding_transcript_variant
Chr13	63243202	T	A	intron_variant	non_coding_transcript_variant
Chr13	63243753	A	T	intron_variant	non_coding_transcript_variant
Chr13	63247390	C	T	intron_variant	non_coding_transcript_variant
Chr13	63248396	C	A	intron_variant	non_coding_transcript_variant
Chr13	63248611	T	C	intron_variant	non_coding_transcript_variant

Chr13	63248986	G	A	intron_variant	non_coding_transcript_variant
Chr13	63249094	A	G	intron_variant	non_coding_transcript_variant

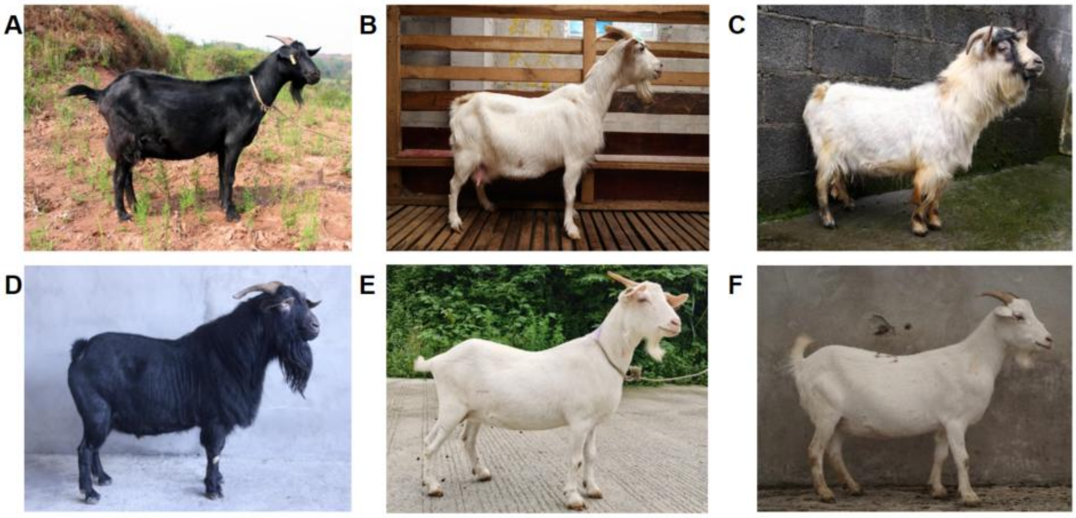

Figure S1. Pictures of six local goat breeds in Chongqing. Note: A is Dazu black goat; B is Banjiao goat; C is Youzhou black goat; D is Yudong black goat; E is Hechuan white goat; F is Chuandong white goat.
